# Hoxa9 compensates for the absence of Hoxc9 in suppressing limb-type motor neurons in sharks

**DOI:** 10.1186/s40851-026-00264-9

**Published:** 2026-02-14

**Authors:** Yuumi Yoshioka, Aoi Shinkai, Masaki Mizutani, Reiko Yu, Toru Kawanishi, Akane Kawaguchi, Shigehiro Kuraku, Mikiko Tanaka

**Affiliations:** 1 Department of Life Science and Technology, Institute of Science Tokyo, B-17, 4259 Nagatsuta-cho, Midori-ku, Yokohama, 226-8501 Japan; 2 Molecular Life History Laboratory, National Institute of Genetics, Mishima, 411-8540 Japan; 3 Department of Genetics, Sokendai (Graduate University for Advanced Studies), Mishima, 411-8540 Japan

**Keywords:** Paired appendages, Innervation, Evolution

## Abstract

**Background:**

The transition from fins to limbs in vertebrates required a novel organization of spinal motor neurons to coordinate limb muscle activation. In amniotes, Hoxc9 represses lateral motor column (LMC) identity at thoracic levels, restricting limb-innervating Foxp1^+^ motor neurons to brachial and lumbar levels. In elasmobranchs, however, the genomic organization of HoxC genes has undergone extensive modifications, and Foxp1^+^ LMC-like neurons have been identified at paired-fin levels in some elasmobranch species lacking *Hoxc9*. These observations suggest that alternative mechanisms regulate motor neuron fate in chondrichthyans, particularly in sharks, although the responsible factors remain unclear.

**Results:**

To identify the mechanism underlying this suppression, we examined Foxp1 and Hox gene expression in chicken and cloudy catshark (*Scyliorhinus torazame*) embryos. In chickens, Foxp1^+^ LMC neurons initially appeared at all rostrocaudal levels but became restricted to paired-limb levels through Hoxc9-mediated repression. In contrast, in cloudy catsharks, which lack *Hoxc9*, Foxp1 was downregulated at inter-fin levels where *Hoxa9* is expressed. Sequence analysis revealed that the *Foxp1* modulatory domain (MD), associated with LMC repression, is highly conserved in *Hoxa9* across all examined chondrichthyan species. *Hoxc9* genes are absent in most sharks but retained in rays and holocephalans, and these retained copies preserved the *Foxp1* MD. Functional analysis in chicken embryos demonstrated that cloudy catshark *Hoxa9* represses LMC identity and promotes preganglionic column (PGC) fate, similar to *Hoxc9* in amniotes.

**Conclusions:**

These findings suggest that conserved Hox9-dependent mechanisms restrict Foxp1^+^ motor neurons at thoracic levels in sharks. In the absence of *Hoxc9*, cloudy catshark *Hoxa9* retains the ability to repress Foxp1 and promote PGC fate, thereby contributing to the organization of motor innervation at paired-fin levels.

**Supplementary information:**

The online version contains supplementary material available at 10.1186/s40851-026-00264-9.

## Introduction

Transition from fins to limbs in vertebrates necessitated the establishment of a novel organization of spinal motor neurons to coordinate the complex activation of limb muscles. The emergence of limb-innervating circuits in terrestrial vertebrates facilitated the diversification of behaviors and habitats [1, 2].

In tetrapods, the spinal motor neurons are organized into discrete columns at specific axial levels. Lateral motor columns (LMCs) at the brachial and lumbar levels primarily innervate limb muscles [3, 4], whereas preganglionic column (PGC) neurons at thoracic levels innervate sympathetic ganglia, and hypaxial motor column (HMC) neurons control body wall muscles [5, 6]. In chickens, PGC neurons reside medially as the Column of Terni (CT) neurons [5]. Median motor columns (MMCs), in contrast, span all rostrocaudal levels and innervate dorsal axial musculature [5, 7]. Limbless vertebrates such as lampreys lack LMCs and rely on MMC-like neurons for trunk muscle innervation and locomotion [1, 8, 9].

Hox gene expression in the spinal cord correlates with the axial patterning of motor columns [10, 11]. Hox6 and Hox10 proteins localize to brachial and lumbar levels, respectively, whereas Hoxc9 specifies PGC and HMC neurons at thoracic levels [10–13]. The forkhead transcription factor Foxp1, regulated by Hox proteins, plays a crucial role in defining LMC neurons that innervate limb muscles [14, 15].

In mice, Foxp1 is initially expressed at all rostrocaudal levels but becomes downregulated at thoracic levels by Hoxc9, redirecting LMC neurons toward PGC and HMC fates [16]. Similarly, in chickens, low Foxp1 expression at thoracic level leads to the segregation of motor neurons into CTs (PGCs) and HMCs [15]. The role of Hoxc9 in repressing LMC fate and promoting PGC identity is also shared by Hoxa9 in mice and chickens [10, 13, 16]. A conserved Foxp1-repressive motif, termed the Foxp1 modulatory domain (MD), has been identified in both proteins [16]. Notably, the Foxp1 MD sequence is present in Hoxc9 of the elephant shark *Callorhinchus milii*, where it converts brachial LMC neurons to PGC neurons in chicken spinal cord [16].

The limb-innervating system is believed to have evolved alongside the emergence of terrestrial vertebrates [17–19]. Foxp1^+^ limb-projecting neurons were long presumed to be a feature limited to tetradpods; however, Jung et al. (2018) demonstrated that Foxp1^+^ motor neurons are present at pectoral and pelvic fin levels in the little skate *Leucoraja erinacea* and the small-spotted catshark *Scyliorhinus canicula* [20]. Importantly, Hox cluster in elasmobranchs (sharks and rays) has undergone extensive lineage-specific erosion and structural divergence [21, 22]. As a result, the presence and integrity of individual HoxC genes, including Hoxc9, vary among species.

Here we investigate the evolutionary development of motor innervation toward paired appendages. We show that in both chicken and cloudy catshark embryos, LMC neurons expressing high levels of Foxp1 are initially present at all rostrocaudal levels but later transition to low Foxp1 expression at thoracic levels. In chicken embryos, Foxp1 suppression occurs in thoracic motor neurons expressing Hoxc9, whereas in cloudy catsharks, which lack Hoxc9, Foxp1 is suppressed in inter-fin motor neurons expressing Hoxa9. *Foxp1* MD sequences are conserved in Hoxa9 across all examined chondrichthyan species. In contrast, Hoxc9 genes were identified in only two shark species, with limited MD homology, while Foxp1 MD is conserved in the Hoxc9 of rays and holocephalans. Functional analysis further revealed that cloudy catshark Hoxa9 represses brachial LMC fate and promotes PGC fate in the chicken spinal cord. Together, these findings raise the possibility that conserved Hox9-dependent mechanisms contribute to the organization of motor innervation of paired appendages in sharks.

## Materials and methods

### Animals

Chicken (*Gallus gallus*) eggs were incubated at 38 °C in a humidified incubator until the desired Hamburger-Hamilton (HH) stage [23] was reached. Cloudy catshark (*S. torazame*) eggs were incubated at 12–16 °C in seawater and staged according to [24].

### Immunohistochemistry

Section immunostaining was performed as described [25]. Briefly, chicken and catshark embryos were fixed overnight with 4% paraformaldehyde in phosphate-buffered saline (PBS), and incubated with 30% sucrose overnight, embedded in OCT compound (Tissue-Tek, Sakura Finetek), frozen and sectioned at 12–16 μm. Cryosections were blocked for 1 hour with 2% bovine serum albumin in PBS, and incubated overnight with 1:50 (for chicken) or 1:100 (for catshark) anti-Islet1/2 (Isl1/2) antibody (39.4D5-s, Developmental Studies Hybridoma Bank) [26], 1:100–1:200 anti-FOXP1 antibody (ab16645, abcam, for chicken; ab93807, abcam, for catshark), anti-Hoxc9 antibody (5B5-2, Developmental Studies Hybridoma Bank) [27], 1:200 anti-GFP (MSFR101910, Nittobo Medical), or 1:1000 anti-Cleaved Caspase-3 antibody (Cat#9664, Cell Signaling) at 4 °C. Sections were washed and incubated overnight with 1:1000 goat anti-rabbit IgG Alexa Fluor-488 conjugated antibody (ab150077, abcam), goat anti-rabbit IgG Alexa Fluor-568 conjugated antibody (Cat#A11011, Invitrogen), goat anti-mouse IgG Alexa Fluor-488 (Cat#A11001, Invitrogen), or goat anti-mouse IgG Cy5 (ab6563, abcam), and 1:500 goat anti-rabbit IgG Alexa Fluor-633 (Cat#A21070, Invitrogen) at 4 °C. After washing, sections were stained with 1:1000 Hoechst and mounted using Vectashield (Vector Laboratories).

### Sequence comparison of Hox9 paralogs

Amino acid sequences of Hox9 paralogs were collected from publicly available databases for representative vertebrate species (Supplementary Table S1). Amino acid sequences were aligned using MAFFT v7.475. The Hox repression domain (RD) and the adjacent C-terminal Foxp1 modulatory domain (MD) were identified based on previously defined domain boundaries [16]. Conservation of the MD was assessed by inspection of the aligned regions.

### Probe synthesis and in situ hybridization

Total RNA was extracted from stage 25 small-spotted catshark *S. canicula*, and stage 24 chicken embryos. cDNA was synthesized by reverse transcription and used as a template for PCR. To amplify *S. canicula Hoxa9,* and chicken *Bmp5* fragments, primers were designed based on *S. canicula Hoxa9* (GenBank FQ032658.1) [28], and chicken *Bmp5* (ENGALT0015019957.1 BMP5-201), respectively, with the sequences complementary to the ends of the linealized pBluescript II SK(+): *S. canicula Hoxa9* (535 bp), 5′-ATCGATAAGCTTGATATGTCGACATCAGGAACTATC-3′ and 5′-CTGCAGGAATTCGATCCATGTGTAGCTTATCTGC-3′;; chicken *Bmp5* (549 bp), 5′- ATCGATAAGCTTGATGGGGACGGTCGAAGTATCAA −3′ and 5′- CTGCAGGAATTCGATTTCATCCTAGTGGCAGCCAC −3′. PCR fragments were cloned into the *Eco*RV site of the pBluescript II SK(+) using the In-Fusion HD Cloning Kit (Clontech), and used as templates for riboprobe synthesis. Section in situ hybridization was carried out as previously described [29].

### In ovo DNA electroporation

For pCAGGS-*S. torazame Hoxa9* (*StHoxa9*), double-stranded DNA fragments (gBlocks Gene Fragments) encoding the open reading frame of *S. torazame Hoxa9* (GenBank MF398244.1) with a Kozak sequence were purchased from Integrated DNA Technologies, and inserted into the *Xho*I site of pCAGGS vector [30] using the In-Fusion reaction (Clontech). Plasmid was mixed with 3% Fast Green and co-electroporated with pCAGGS-*EGFP* (a gift from Dr. Miyazaki and Dr. Ogura) into the neural tube of HH14 chicken embryos. The plasmid solution was injected into the neural tube, followed by electroporation using CUY21 EDIT II (BEX Co., Ltd.) with five pulses of 20 V, 20 ms. The final concentrations of the plasmid solutions were as follows: pCAGGS-*EGFP*, 3.5 mg/ml; pCAGGS-*StHoxa9*, 3.5 mg/ml. Embryos were harvested and fixed at HH27, three days post-electroporation.

### Images

Images were captured using an LSM780 confocal microscope (Zeiss), a upright microscope BX61WI (Olympus) or a single-lens reflex camera (DP74, Olympus). Pseudo color images were generated using Zen black software (Zeiss).

## Results

### Spatial and temporal expression of Hoxc9 and Foxp1 in developing chicken spinal cord

**Fig. 1:**
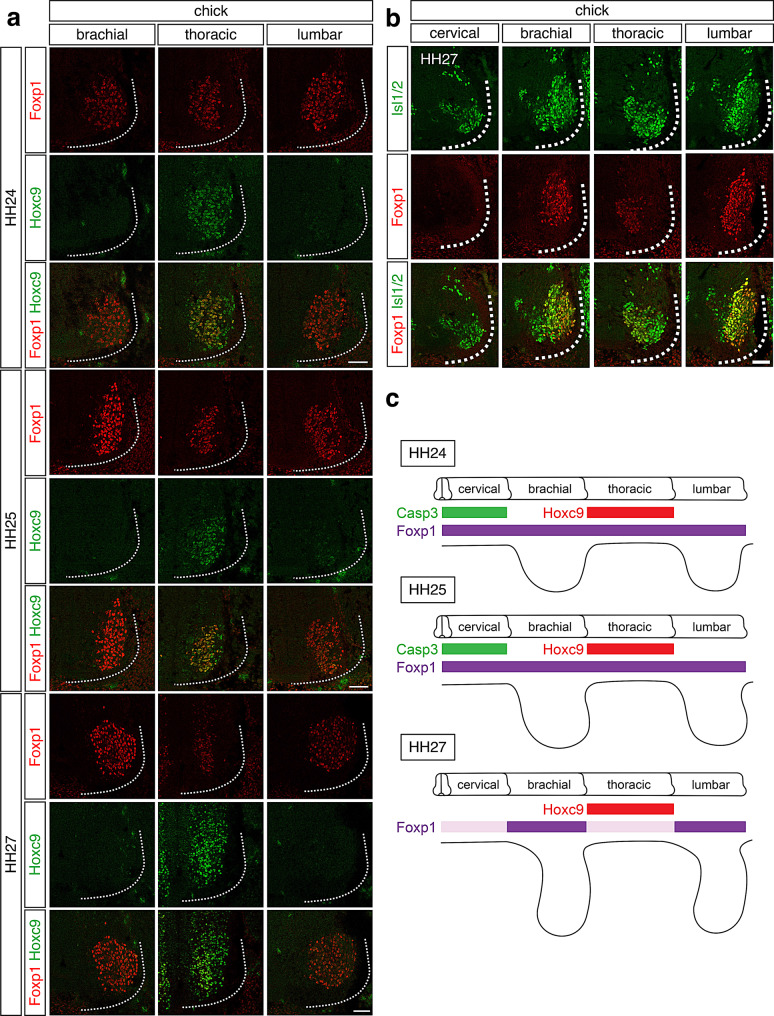
Distribution of Foxp1, Hoxc9 and Isl1/2 in spinal cords of chick embryos. **a**. High levels of Foxp1 were detected at brachial, thoracic and lumbar MNs at HH24 (*n* = 3) and HH25 (*n* = 3) and lowered at thoracic level by HH27 (*n* = 4). Hoxc9 proteins were detected in Foxp1^+^MNs at thoracic level from HH24 to HH27. Scale bars, 50 μm. **b**. Foxp1^+^ MNs were removed at cervical level, whereas Foxp1 proteins were lowered in MNs at thoracic level at HH27 (*n* = 3). Scale bars, 50 μm. **c**. Summary of MN columnar organization in developing chicken embryos

In mice, Foxp1 expression initially appears throughout the spinal cord at all rostrocaudal levels but is later downregulated at thoracic levels by Hoxc9 as embryogenesis proceeds [16]. To determine whether a similar process occurs in chickens, we analyzed the spatial and temporal expression of Foxp1 and Hoxc9 in developing chicken spinal cords (Fig. 1a). At HH24, Foxp1 was highly expressed in motor neurons at brachial, thoracic and lumbar levels. By HH25, Foxp1 expression at thoracic levels began to decrease and was nearly absent by HH27 (Fig. 1a). Consistent with findings in mice, Hoxc9 expression persisted at thoracic levels from HH25 to HH27, even after Foxp1 had been downregulated (Fig. 1a). Furthermore, Foxp1 expression co-localized with Hoxc9 at thoracic levels (Fig. 1a). These results indicate that, as in mice, Foxp1 is transiently expressed at thoracic levels and eventually downregulated in Hoxc9^+^ motor neurons in chickens.

In chickens, motor neurons at cervical levels undergo apoptosis during development [31]. To investigate whether apoptosis contributes to the downregulation of Foxp1 at thoracic levels, we examined the distribution of cleaved caspase-3 in chicken embryos at HH24, HH25 and HH27 (Figure S1). Cleaved caspase-3 signals were detected at cervical levels at HH24 and HH25, as previously reported [31]. However, no apoptotic signals were observed at brachial, thoracic, or lumbar levels at any stage examined (Figure S1), suggesting that apoptosis does not contribute to the reduction of Foxp1 expression at thoracic levels by HH27. Analysis of Isl1/2 distribution at HH27 confirmed the absence of Foxp1^+^; Isl1/2^+^ motor neurons at cervical levels, while Isl1/2^+^ motor neurons were present in both Foxp1^+^ neurons at brachial and lumbar levels and Foxp1^-^ neurons at thoracic levels (Fig. 1b). These results further support that apoptosis does not contribute to the downregulation of Foxp1 expression in Hoxc9^+^ motor neurons at thoracic levels.

In summary, Foxp1^+^ motor neurons are initially present at all rostrocaudal levels in early-stage chicken embryos, and Foxp1 expression is eventually downregulated in Hoxc9^+^ motor neurons at thoracic levels (Fig. 1c), consistent with observations in mice [16].

### Spatiotemporal expression of Foxp1 in developing cloudy catshark spinal cord

**Fig. 2:**
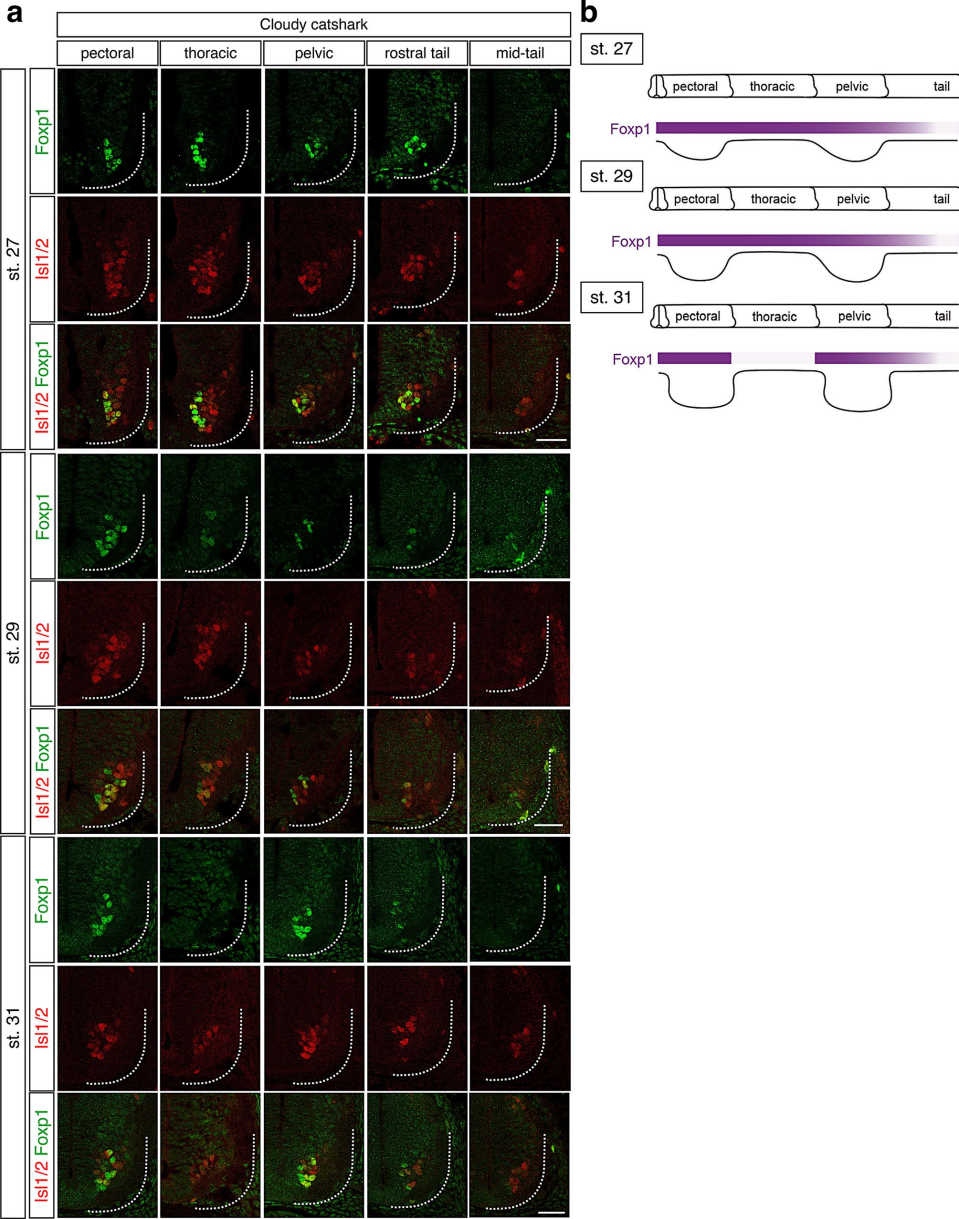
Distribution of Foxp1 and Isl1/2 in spinal cords of cloudy catshark embryos. **a**. Foxp1 protein was detected in MNs at pectoral, thoracic (inter-fin), pelvic and tail levels at stage 27 (*n* = 3) and 29 (*n* = 3). By stage 31, Foxp1 signals were reduced or undetectable at thoracic and mid-tail levels (*n* = 3). Scale bars, 50 μm. **b**. Summary of the distribution of Foxp1^+^ MNs from pectoral to mid-tail levels in cloudy catshark embryos at stages 27, 29 and 31

A recent study showed that, in cloudy catshark (*Scyliorninus torazame*) embryos, Foxp1^+^ motor neurons are present at pectoral and pelvic fin levels but are absent or rare at inter-fin or tail levels at stage 31 [20]. To elucidate the developmental mechanism underlying this restricted distribution, we examined the spatial and temporal distribution of Foxp1 in the cloudy catshark spinal cords at stages 27, 29 and 31 (Fig. 2a). At stages 27 and 29, Foxp1 signals were detected in Isl1/2^+^ motor neurons across all rostrocaudal levels. By stage 31, Foxp1 signals were markedly reduced at inter-fin levels, while remaining detectable at pectoral and pelvic fin levels (Fig. 2a). These observations indicate that Foxp1 is broadly present throughout the spinal cord at early stages and later becomes selectively reduced at inter-fin levels during development (Fig. 2b), resembling the overall temporal pattern observed in chicken and mouse embryos.

To investigate whether apoptosis contributes to the reduction of Foxp1 signals, we examined the distribution of cleaved caspase-3 in cloudy catshark embryos at stages 27 and 31 (Figure S2). Cleaved caspase-3-positive cells were observed in dorsal tissues adjacent to the spinal cord at stage 31 (Figure S2a). However, no apoptotic signals were detected within the spinal cord at any rostrocaudal levels at either stage examined (Figure S2b). These findings suggest that apoptosis does not account for the reduction of Foxp1 expression at inter-fin levels.

### Complementary expression patterns for Hoxa9 and Foxp1 in the cloudy catshark spinal cord

In mouse and chicken embryos, Hoxc9 is known to play a central role in spinal motor neuron organization by restricting Foxp1^+^ LMC fate at thoracic levels [16]. In cloudy catsharks embryos, Foxp1^+^ motor neurons similarly become reduced at inter-fin levels during development. However, the *Hoxc9* gene is absent from the cloudy catshark genome [21]. Previous studies have shown that murine *Hoxa9* can convert brachial LMC neurons into PGC neurons, a function comparable to *Hoxc9* [10, 13, 16]. This activity is mediated by a conserved *Foxp1* modulatory domain (MD) [16], which is also present in *Hoxc9* of the holocephalan *C. milii*, where it confers LMC-to-PGC conversion activity in chicken spinal cord [16].

**Fig. 3:**
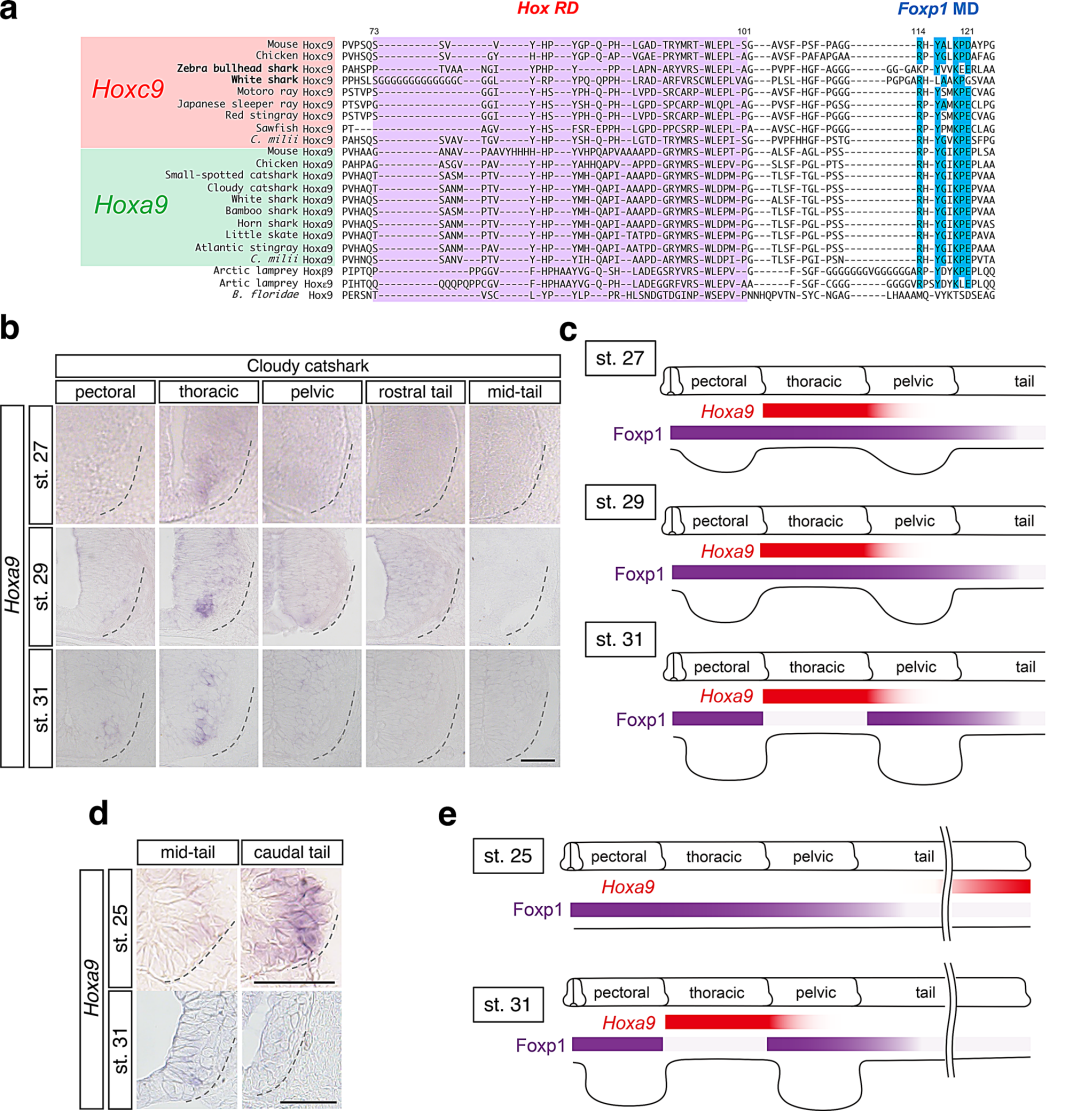
Expression of *Hoxa9* in spinal cords of cloudy catshark embryos. **a**. *Hox* repression domains (*Hox* RD; purple) and *Foxp1* modulatory domains (Foxp1 MD; blue) [16] after the alignment of Hox9 proteins from multiple chordate species. Note that *Foxp1* modulatory domains are collapsed in Hoxc9 from two shark species (zebra bullhead shark and white shark), but conserved among Hoxa9 from all chondrichthyan species examined. **b**. Expression of *Hoxa9* in spinal cords of stage 27 (*n* = 3), 29 (*n* = 3) and 31 (*n* = 3) cloudy catshark embryos. *Hoxa9* expression appeared at thoracic (inter-fin) level from stage 27 and became intensified at the same level by stage 29. A scale bar, 50 μm. **c**. Summary of MN columnar organization from pectoral to mid-tail levels in stages 27, 29 and 31 cloudy catshark embryos. **d**. Expression of *Hoxa9* in spinal cords at mid-tail to caudal-tail levels in stage 25 (*n* = 3) and 31 (*n* = 3) cloudy catshark embryos. Scale bars, 50 μm. **e**. Summary of MN columnar organization from pectoral to caudal-tail levels in stages 25 and 31 cloudy catshark embryos

To examine the conservation of the *Foxp1* MD among elasmobranchs, we aligned the *Hox* repression domain (RD) and the *Foxp1* MD−located C-terminal to the *Hox* RD−in Hoxc9 and Hoxa9 proteins from multiple chondrichthyan species (Fig. 3a). Hoxa9 sequences from seven elasmobranch species, including cloudy catshark, small-spotted catshark, white shark, horn shark, bamboo shark, little skate, red stingray, as well as a holocephalan, *C. milii,* all retained the *Foxp1* MD. In contrast, *Hoxc9* gene was identified in only two shark species (white shark and zebra bullhead shark), but was present in all examined ray species (sawfish, red stingray, motoro ray, Japanese sleeper ray) except members of the family *Radidae*, as well as in *C. milii* (Fig. 3a). The *Foxp1* MD was conserved in Hoxc9 proteins of all examined rays and a holocephalan *C. milii*, whereas it showed substantial divergence in the two shark species analyzed, indicating differences in Hoxc9 sequence conservation among chondrichthyan lineages.

We next examined the spatial expression of *Hoxa9* in developing cloudy catshark embryos. At stages 27, 29 and 31, *Hoxa9* transcripts were detected in the ventral spinal cord at inter-fin (thoracic) levels, with higher intensity observed by stage 31 (Fig. 3b, c). In contrast, *Hoxa9* expression was minimal at mid-tail levels, where Foxp1 signals were also absent from stages 27 to 31 (Fig. 2). To assess whether Hoxa9 expression precedes the reduction of Foxp1 signals, we examined earlier developmental stages. At stage 25, *Hoxa9* was detected from the mid-tail to caudal tail levels, with stronger expression caudally, while no expression was observed from pectoral to rostral-tail levels (Figure S3). By stage 31, *Hoxa9* expression in the caudal tail region was no longer detected (Fig. 3d, e). Together, these results show that *Hoxa9* and Foxp1 exhibit complementary spatiotemporal expression patterns in the cloudy catshark spinal cord, with reduced Foxp1 signals observed following the onset of *Hoxa9* expression.

### Cloudy catshark Hoxa9 retains the repressive activity

**Fig. 4:**
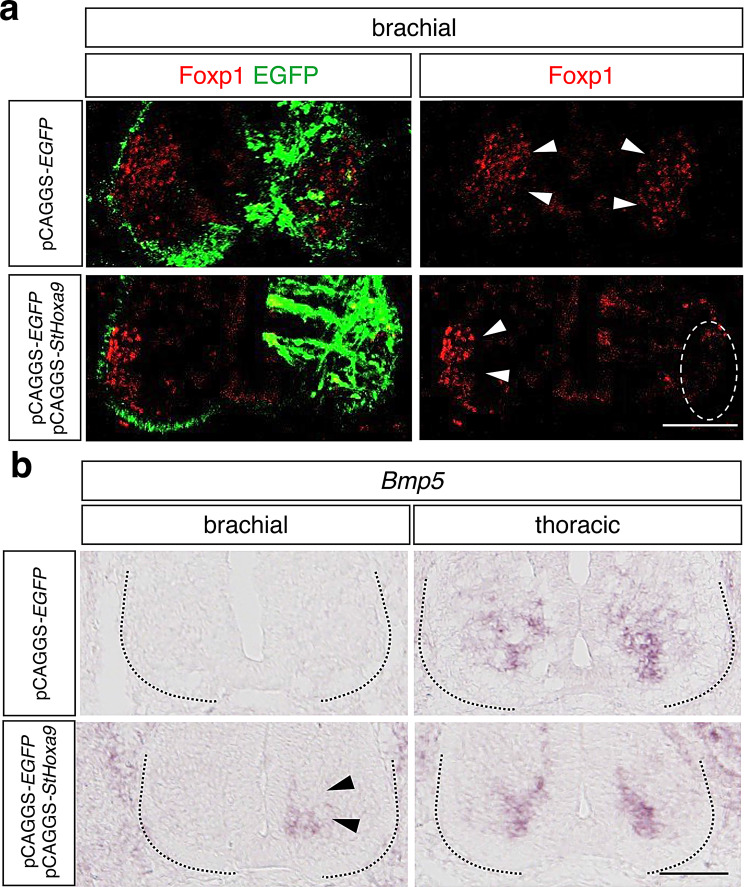
Functional analysis of cloudy catshark *Hoxa9* in the spinal cord of chicken embryos. **a**. Foxp1 distribution in the spinal cord at the brachial level of chicken embryos three days after overexpression of control pCAGGS-*EGFP* or pCAGGS-*EGFP* together with pCAGGS-*StHoxa9* on the right side. Note that Foxp1 signals (arrowheads) were downregulated in the region where both pCAGGS-*EGFP* and pCAGGS-*StHoxa9* were electroporated (a dotted circle; *n* = 3), whereas no changes were observed in the region electroporated with pCAGGS-*EGFP* (*n* = 3). **b**. *Bmp5* expression in the spinal cord at the brachial and thoracic levels of the same embryos. Ectopic *Bmp5* expression (arrowheads) was induced in the region electroporated with both pCAGGS-*EGFP* and pCAGGS-*StHoxa9* (*n* = 3), whereas no changes were observed in the region electroporated with pCAGGS-*EGFP* (*n* = 3). Scale bars, 100 μm. Panels in Fig. 4 awere flipped horizontally

The complementary expression patterns of *Hoxa9* and Foxp1 prompted us to test whether cloudy catshark Hoxa9 possesses Foxp1-repressive activity. To address this, we misexpressed cloudy catshark *Hoxa9* (*StHoxa9*) at the brachial level of chicken embryos using in ovo electroporation (Fig. 4a). Electroporation of the control vector of pCAGGS-*EGFP* alone did not alter Foxp1 expression, whereas co-electroporation of pCAGGS-*StHoxa9* with pCAGGS-*EGFP* resulted in a clear reduction of Foxp1 signals on the electroporated side (Fig. 4a), indicating that cloudy catshark Hoxa9 is capable of repressing LMC specification.

To determine whether cloudy catshark *Hoxa9* also promotes PGC fate, we examined *Bmp5* expression following electroporation (Fig. 4b). Electroporation of pCAGGS-*EGFP* alone did not induce ectopic *Bmp5* expression at the brachial level. In contrast, co-electroporation of pCAGGS-*StHoxa9* with pCAGGS-*EGFP* induced ectopic *Bmp5* expression on the electroporated side (Fig. 4b). No changes in *Bmp5* expression were observed at the thoracic levels following electroporation of either pCAGGS-*EGFP* alone or pCAGGS-*StHoxa*9 with pCAGGS-*EGFP* (Fig. 4b). Together, these findings demonstrate that cloudy catshark *Hoxa9* retains the ability to repress LMC fate and promote PGC fate in the chicken spinal cord.

## Discussion

In this study, we show that motor neurons expressing high levels of Foxp1 are initially present at all rostrocaudal levels in both chicken and cloudy catshark embryos, but later become reduced at thoracic and inter-fin levels. Cloudy catsharks lack Hoxc9, yet exhibit Hoxa9 expression in regions where Foxp1 signals are reduced. Functional analyses further demonstrated that cloudy catshark Hoxa9 is capable of repressing LMC specification and promoting PGC fate in the chicken spinal cord. Together, these findings indicate that conserved Hox9-dependent mechanisms underlin the restriction of limb-type motor neurons across vertebrates, and that in sharks lacking Hoxc9, Hoxa9 can assume a prominent role in this process.

Hoxc9 plays a key role in restricting limb-innervating motor neurons to brachial and lumbar levels by gradually suppressing thoracic Foxp1 expression at thoracic levels as mouse embryogenesis proceeds [10, 13, 16]. Consistent with this, Foxp1 is initially present at all rostrocaudal levels in chicken embryos and later becomes reduced in Hoxc9^+^ motor neurons at thoracic levels, as we confirmed by examining its temporal dynamics. Unlike cervical LMC neurons, which are eliminated by apoptosis [31], thoracic motor neurons showed no apoptotic signals. Because Isl1/2 signals were retained in Foxp1^low^, Hoxc9^+^ thoracic neurons, these neurons are likely redirected toward PGC fates, as described in mice [14, 16]. In tetrapods, Foxp1 is expressed in and required for PGC development, although Foxp1 expression alone is not sufficient to specify limb-innervating motor neuron identity. HMC and PGC motor neurons are present at thoracic levels in HH30 chicken embryos [15], supporting the idea that Foxp1^+^ LMC neurons initially present throughout the spinal cord are progressively redirected toward thoracic fates as Hoxc9-mediated repression proceeds. This temporal program—broad early Foxp1 expression followed by Hox-mediated repression at non-limb levels—appears to be conserved among amniotes, and possibly across other terrestrial vertebrates, as previously suggested [16, 20].

By comparing amniotes with chondrichthyans, our findings demonstrate that a similar developmental program restricting Foxp1^high^ motor neurons to paired-fin levels is already present in the chondrichthyan lineage. Previous studies proposed that ancestral Hox9 proteins may have originally promoted LMC identity, and that Foxp1-repressive and PGC-promoting activities emerged through the evolution of the Foxp1 MD domains [16]. Consistent with this view, *Hoxc9* of *C.milii* and *Hoxa9* of the little skate *L. erinacea* can convert brachial LMC neurons into PGC neurons in chicken embryos [16, 20]. Importantly, the status of Hoxc9 within elasmobranchs is heterogeneous, ranging from complete loss to retention of highly diverged copies, whereas rays and holocephalans consistently retain a conserved Hoxc9. Genome assemblies and transcriptome-based surveys have documented extensive reduction and fragmentation of the HoxC cluster in elasmobranchs, with marked variation among species [21, 32]. Our analyses show that the Foxp1 MD is conserved in Hoxa9 across chondrichthyans and in Hoxc9 of most rays and holocephalans. In contrast, Hoxc9 genes were identified only in two shark species, both of which exhibit reduced conservation of the Foxp1 MD. In cloudy catsharks, *Hoxa9* expression is prominent at inter-fin and caudal-tail levels where Foxp1 signals are reduced, and functional assays confirmed that cloudy catshark *Hoxa9* retain the ability to repress LMC fate and promotes PGC fate. Together, these observations suggest that in sharks lacking functional *Hoxc9*, the conserved Foxp1-repressive activity of *Hoxa9* plays a prominent role in restricting LMC neurons to paired-fin levels.

An important caveat concerns the mechanism underlying Foxp1 repression at caudal spinal levels. Although Hoxa9 expression precedes the reduction of Foxp1 signals in the caudal spinal cord of cloudy catshark embryos, many Hox genes show transient or overlapping expression in posterior spinal regions. In particular, more posterior Hox paralogs, such as Hox11-13 genes, may also contribute to Foxp1 repression at caudal levels, either directly or indirectly through repression of more rostral Hox genes that promote Foxp1 expression. Therefore, while our data are consistent with a role for Hoxa9 in Foxp1 repression at inter-fin levels, we cannot exclude the possibility that distinct Hox-dependent mechanisms operate at more posterior axial positions.

The little skate *Leucoraja* provides an informative exception. In- *Leucoraja, Hoxa9* expression overlaps with Foxp1 at caudal pectoral fin levels [20], and expansion of pectoral LMC neurons has been attributed to the absence of Hoxc9 [20]. However, our broader survey revealed that most rays and skates retain *Hoxc9* with conserved *Foxp1* MD domains, with *Leucoraja* representing a notable exception. In line with these observations, *Leucoraja Hoxa9* can attenuate Foxp1 expression in chicken brachial neurons [20], whereas mouse *Hoxc9* failed to repress Foxp1 in skate embryos [20]. Jung et al. (2018) therefore proposed that skates may lack accessory factors required for effective *Hox9*-mediated Foxp1 repression.

Phylogenetic comparisons provide additional insight into the ancestral state of Hox9 function. In the common ancestor of chondrichthyans, both *Hoxa9* and *Hoxc9* likely retained *Foxp1* MD domains. After the divergence of holocephalans and elasmobranchs, the latter lineages experienced a marked relaxation of structural constraint on the HoxC cluster. In elasmobranchs, the HoxC cluster is often considerably longer than 100 kb and is extensively invaded by repetitive elements, with a reduced number of Hox genes, in contrast to many other jawed vertebrates in which the cluster remains compact (~100 kb) and relatively free of transposons [33]. As a consequence, some shark species lost *Hoxc9* or retained only *Hoxc9* with reduced *Foxp1* MD conservation, whereas *Hoxa9* maintained Foxp1-repressive activity (Fig. 3a). It remains unresolved whether *Hoxa9* originally repressed Foxp1 at paired-fin levels prior to the split between cyclostomes and gnathostomes, or whether *Hoxc9* first acquired this ability in the tetrapod lineage and was later functionally compensated by *Hoxa9* in certain elasmobranchs. Notably, lamprey Hoxβ9 and Hoxδ9 retain *Foxp1* MD-like regions and show weak Foxp1-repressive and PGC-promoting activity in chicken spinal neurons [16], whereas amphioxus Hox9 lacks the *Foxp1* MD and promotes LMC fate [16]. These findings support the idea that a Foxp1-repressive MD may have been present in ancestral Hox9 proteins of early vertebrates. The emergence and differential deployment of Hox9-mediated repression of Foxp1 at thoracic/inter-fin levels likely constituted a critical step in the evolution of neural circuits controlling paired appendage locomotion.

## Conclusion

Our study demonstrates that the conserved Hox9-dependent mechanisms underlie the restriction of limb-type motor neurons across vertebrates. In the absence of *Hoxc9*, cloudy catshark *Hoxa9* retains a conserved ability to repress *Foxp1* and promote PGC fate, thereby playing a prominent role in restricting limb-type motor neurons at paired-fin levels. Comparative sequence analyses further indicate that while *Hoxa9* broadly retains the *Foxp1* modulatory domain across chondrichthyans, *Hoxc9* has been lost or structurally diverged in many shark lineages. These findings underscore the ancestral importance of Hox9-dependent regulatory mechanisms in restricting limb-type motor neurons associated with paired appendages.

## Electronic supplementary material

Below is the link to the electronic supplementary material.

Supplementary Material 1: Figures S1–S3. Distribution of cleaved caspase-3 and Hoxa9 expression in chicken and cloudy catshark embryos.

Supplementary Material 2: Table S1. Genome information of species used in this study.

## Data Availability

All sequence data analyzed in this study are publicly available.
