# Supplementary material for: Hoxa9 compensates for the absence of Hoxc9 in suppressing limb-type motor neurons in sharks
Source: Zoological Lett. 2026 Feb 14;12:8. doi: 10.1186/s40851-026-00264-9 (PMC13097845; doi:10.1186/s40851-026-00264-9)
Supplement: Supplementary file 1 — Supplementary Material 1: Figures S1–S3. Distribution of cleaved caspase-3 and Hoxa9 expression in chicken and cloudy catshark embryos. [file 40851_2026_264_MOESM1_ESM.pdf]

Additional File 1

Hoxa9 compensates for the absence of Hoxc9 in suppressing limb-type motor neurons in sharks

Yuumi Yoshioka[1,†], Aoi Shinkai[1,†], Masaki Mizutani[1], Reiko Yu[1], Toru Kawanishi[1], Akane Kawaguchi[2,3], Shigehiro Kuraku[2,3], and Mikiko Tanaka[1,*]

[1]Department of Life Science and Technology, Institute of Science Tokyo, B-17, 4259 Nagatsuta-cho, Midori-ku, Yokohama, Japan.

[2] Molecular Life History Laboratory, National Institute of Genetics, Mishima, Japan

[3] Department of Genetics, Sokendai (Graduate University for Advanced Studies), Mishima, Shizuoka, 411-8540, Japan

[†]These authors contributed equally to this work.

*Correspondence: tanaka.m.3434@m.isct.ac.jp

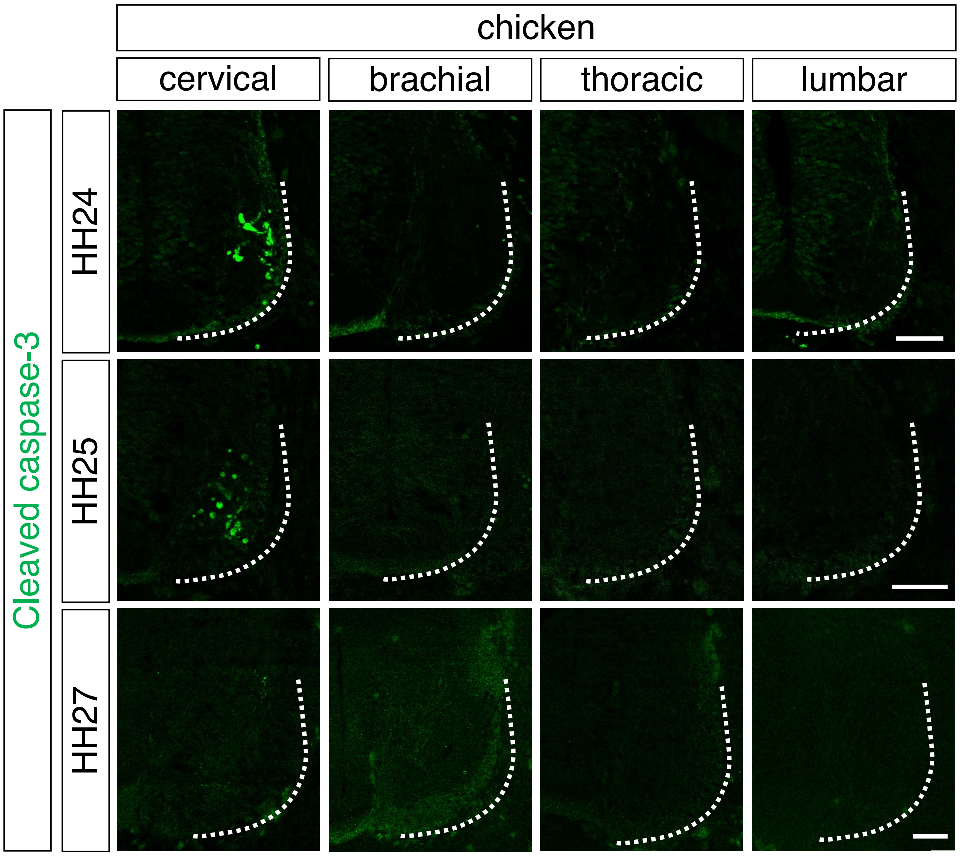

Fig. S1. Distribution of cleaved caspase-3 in MNs of developing chicken embryos. Cleaved caspase-3 signal was detected at cervical level, but not at thoracic level of stages 24 (n=3), 25 (n=3) or 27 (n=3) chicken embryos. Scale bars, 50 µm.

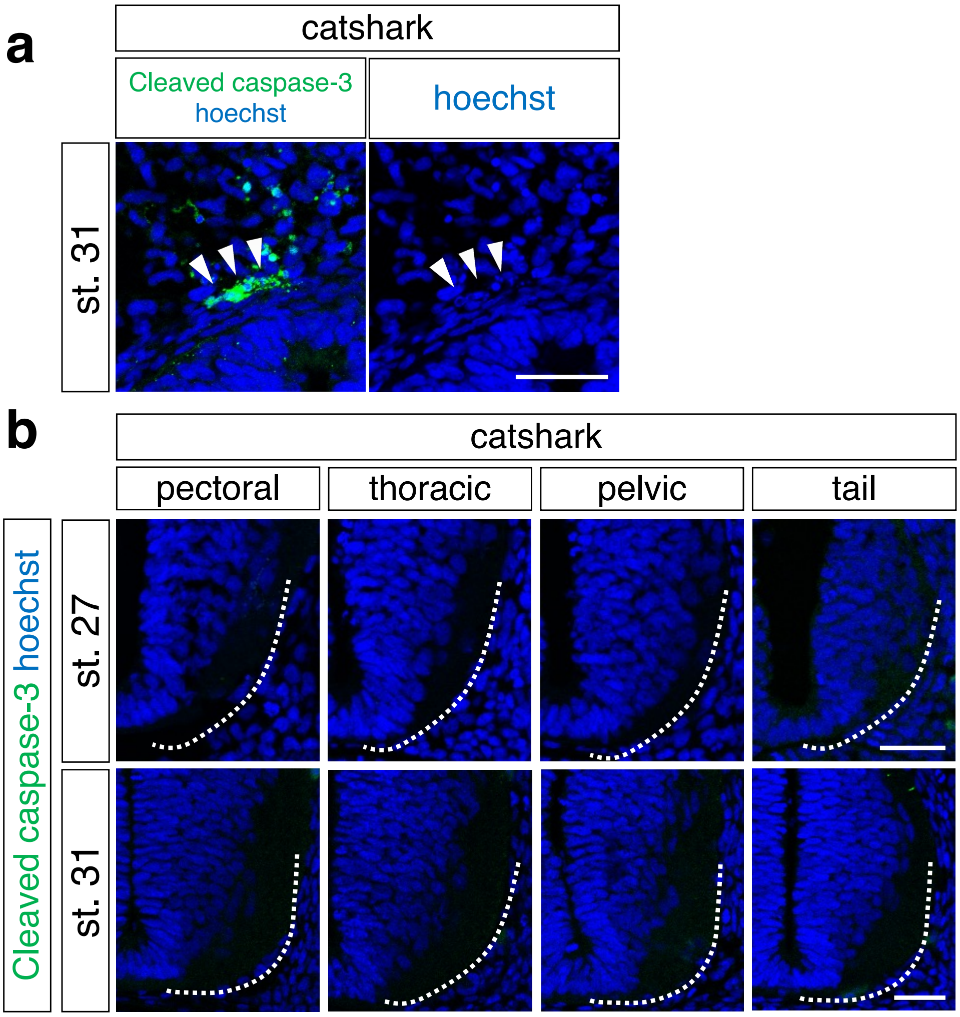

Fig. S2. Distribution of cleaved caspase-3 in cloudy catshark embryos. **a.** Cleaved caspase-3 signal was detected in dorsal region adjacent to the spinal cord (n=2). **b.** No cleaved caspase-3 signal was detected in MNs at any levels of stage 27 (n=2) or 31 (n=3) cloudy catshark embryos. Scale bars, 50 μm.

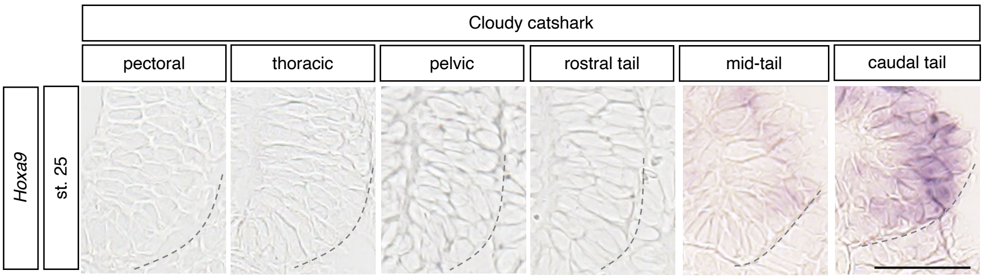

Fig. S3. Expression of *Hoxa9* in stage 25 cloudy catshark embryos. *Hoxa9* expression in the spinal cord of a stage 25 cloudy catshark embryos (the same embryo shown in Fig. 3d) (n=3). Scale bars, 50 μm.
